# Poly[tetra­butyl­tetra­kis­(μ_2_-hydrogen phenyl­phospho­nato)ditin(IV)]

**DOI:** 10.1107/S1600536812040834

**Published:** 2012-10-06

**Authors:** Modou Sarr, Aminata Diasse-Sarr, Libasse Diop, Kieran C. Molloy, Gabriele Kociok-Kohn

**Affiliations:** a Laboratoire de Chimie Minérale et Analytique, Département de Chimie, Faculté des Sciences et Techniques-Université Cheikh Anta Diop, Dakar, Senegal; b Department of Chemistry, University of Bath, Claverton Down, Bath BA2 7AY, England

## Abstract

In the title compound, [Sn_2_(C_4_H_9_)_4_(C_6_H_6_PO_3_)_4_]_*n*_, the basic unit is a dimer containing two symmetry-related Sn^IV^ atoms bridged by two hydrogenphenylphosphonate anions. This fragment is located about an inversion center, and each Sn^IV^ atom is linked to two other hydrogenphenylphosphonate anions, giving a layered structure parallel to (010). The coordination geometry for the Sn^IV^ atoms is close to octa­hedral. The layers are connected *via* O—H⋯O hydrogen bonds, generating a three-dimensional network. One butyl group is disordered over two sets of sites, with occupancies of 0.49 (2) and 0.51 (2).

## Related literature

For medicinal applications of Sn^IV^ compounds, see: Evans & Karpel (1985 ▶). For the biocidal activity of organotin compounds, see: Molloy *et al.* (1981 ▶). For background to the search for new organotin compounds, see: Holmes *et al.* (1988 ▶); Hadjikakou & Hadjiliadis (2009 ▶). For work in this field carried out by the authors, see: Diassé-Sarr *et al.* (1997 ▶); Sall *et al.* (1992 ▶); Boye & Diassé-Sarr (2007 ▶); Diop *et al.* (2011 ▶).
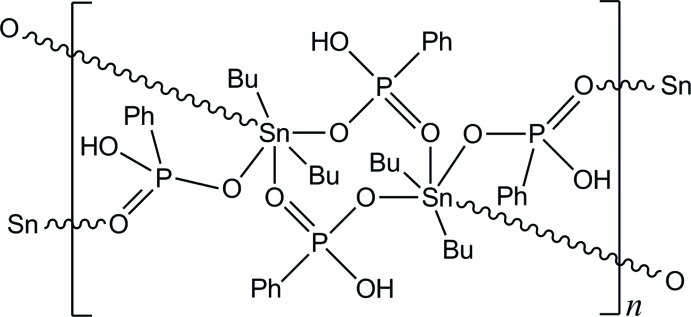


## Experimental

### 

#### Crystal data

[Sn_2_(C_4_H_9_)_4_(C_6_H_6_PO_3_)_4_]
*M* *_r_* = 1094.14
Triclinic, 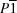

*a* = 11.0258 (3) Å
*b* = 13.8500 (4) Å
*c* = 16.0177 (4) Å
α = 74.074 (1)°
β = 89.742 (1)°
γ = 77.291 (1)°
*V* = 2290.44 (11) Å^3^
*Z* = 2
Mo *K*α radiation
μ = 1.29 mm^−1^
*T* = 150 K
0.30 × 0.25 × 0.10 mm

#### Data collection

Nonius Kappa CCD diffractometer
Absorption correction: multi-scan (*SORTAV*; Blessing, 1995 ▶) *T*_min_ = 0.699, *T*_max_ = 0.882
35391 measured reflections
10394 independent reflections
8145 reflections with *I* > 2σ(*I*)
*R*_int_ = 0.060

#### Refinement

*R*[*F* ^2^ > 2σ(*F* ^2^)] = 0.053
*wR*(*F* ^2^) = 0.142
*S* = 1.05
10394 reflections
570 parameters
4 restraints
H atoms treated by a mixture of independent and constrained refinement
Δρ_max_ = 2.73 e Å^−3^
Δρ_min_ = −1.93 e Å^−3^

### 

Data collection: *COLLECT* (Nonius, 1998 ▶); cell refinement: *DENZO* and *SCALEPACK* (Otwinowski & Minor, 1997 ▶); data reduction: *DENZO* and *SCALEPACK*; program(s) used to solve structure: *SIR97* (Altomare *et al.*, 1999 ▶); program(s) used to refine structure: *SHELXL97* (Sheldrick, 2008 ▶); molecular graphics: *ORTEP-3* (Farrugia, 1997 ▶); software used to prepare material for publication: *WinGX* (Farrugia, 1999 ▶).

## Supplementary Material

Crystal structure: contains datablock(s) I, New_Global_Publ_Block. DOI: 10.1107/S1600536812040834/bh2452sup1.cif

Structure factors: contains datablock(s) I. DOI: 10.1107/S1600536812040834/bh2452Isup2.hkl

Additional supplementary materials:  crystallographic information; 3D view; checkCIF report

## Figures and Tables

**Table 1:** Hydrogen-bond geometry (Å, °)

*D*—H⋯*A*	*D*—H	H⋯*A*	*D*⋯*A*	*D*—H⋯*A*
O3—H3⋯O10^i^	0.87 (2)	1.80 (1)	2.656 (5)	172 (6)
O6—H6⋯O1	0.86 (2)	1.77 (1)	2.628 (5)	172 (6)
O9—H9⋯O4	0.87 (2)	1.83 (2)	2.662 (5)	159 (6)
O12—H12⋯O8^ii^	0.88 (2)	1.78 (2)	2.633 (5)	168 (6)

Symmetry codes: (i) 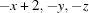; (ii) 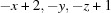.

**Fig. 1.**
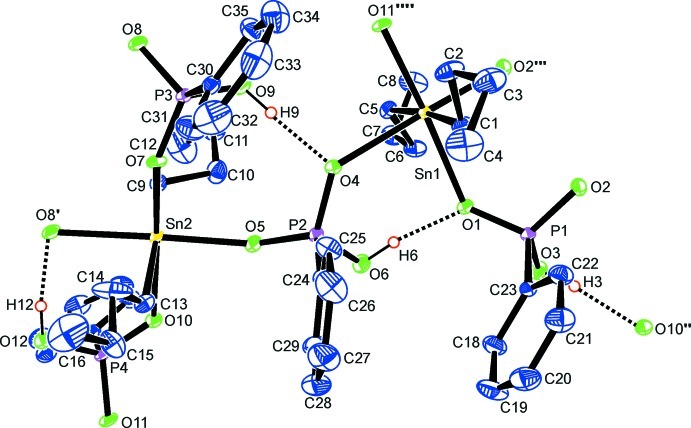
Three dimensional structure showing the hydrogen bonds as dotted lines.

**Fig. 2.**
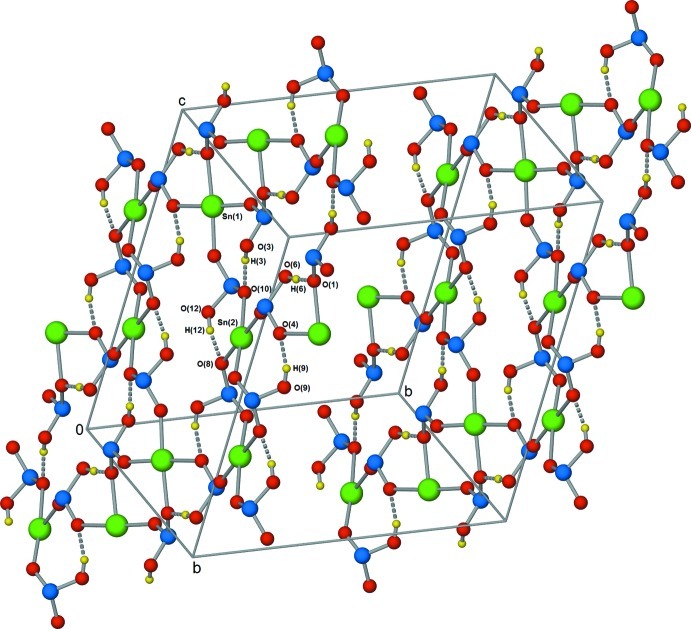
A part of the crystal structure.

## supplementary crystallographic information

### Comment

The interest to synthesize new organotin derivatives is related to their various applications in different fields: agrochemicals, surface disinfectants and marine antifouling paints, *etc*. (Evans & Karpel, 1985); thus many groups have been involved in the search for new organotin compounds (Holmes *et al.*, 1988; Hadjikakou & Hadjiliadis, 2009). Our group has yet published some papers dealing with SnBu_2,_ SnMe_3_ and SnPh_3_-residues containing derivatives (Diassé-Sarr *et al.*, 1997; Sall *et al.*, 1992; Boye & Diassé-Sarr, 2007). In continuation of this work, we have initiated here the study of the interactions between PhPO_3_H_2_ and SnBu_2_Cl_2_ which yielded Sn_2_Bu_4_(PhPO_3_H)_4_ . The phosphorous acids are very important in *in vivo* systems. Phosphorous acids are known for their biocidal activities, as some organotin compounds (Molloy *et al.*, 1981). Combining them seems worthy for having specific derivations allowing a positive combination of that property. This explains our focus on that type of compounds.

The asymmetric unit of the title compound contains two dibutyltin(IV) units and four hydrogen phenylphosphonates, leading to [SnBu_2_(PhPO_3_H)_2_]_2_ formula (Fig. 1). The structure consists to two equivalents Sn1 atoms bridged by two hydrogenophenylphosphonates, generating a dimer. This fragment is located on a inversion center. Each Sn1 atom is linked to two others dimers based on Sn2 atoms, *via* other bridging hydrogenophenylphosphonates (P2, P3, P4), generating a 2D polymer. Hydrogen bonds O—H···O involving the P—OH groups of the hydrogenophenylphosphonates give a 3D crystal structure (fig. 2).

The hydrogen bonds lead to almost equal P—O bond distances [P1—O1: 1.513 (3) Å, P1—O2: 1.516 (3) Å, different of the P—OH bond: P1—O3: 1.563 (3) Å], as reported for dicyclohexylammonium trimethylbis(hydrogen phenylphosphonato)-stannate(IV) (Diop *et al.*, 2011). The geometry around the P atom is a distorted tetrahedron [O1—P1—O2: 113.49 (18)°, O1—P1—C23: 109.6 (2)°]. The sum of the O—Sn—O angles is 360.13° for Sn1 and the C1—Sn1—C5 angle value of 174.37 (18)° indicates some deviation from ideal *trans* octahedral arrangement around the Sn^IV^ ions.

### Experimental

The title compound has been synthesized by allowing PhPO_3_H_2 _(0.2 g) to react with SnBu_2_Cl_2 _(0.1 g) in ethanol (1/1 ratio). The mixture was stirred for two hours and submitted to a slow solvent evaporation at room temperature, giving, after some days, regular colorless crystals suitable for X-ray work (m.p. 463 K).

### Refinement

One butyl group was found to be disordered: atoms C14, C15 and C16 are disordered with C14A, C15A, C16A, and occupancy factors converged to 0.49 (2) and 0.51 (2), respectively. Hydroxyl H atoms (H3, H6, H9 and H12) were found in a difference map and refined freely, although with restrained bond lengths. Other H atoms were placed in idealized positions and refined as riding to their carrier C atoms, with C—H = 0.95 (aromatic CH), 0.98 (methyl CH_3_) or 0.99 Å (methylene CH_2_ groups). Isotropic displacement parameters for H atoms were calculated as *U*_iso_(H) = *xU*_eq_(carrier atom); *x* = 1.2 or 1.5.

### Crystal data

**Table:**

[Sn_2_(C_4_H_9_)_4_(C_6_H_6_PO_3_)_4_]	*Z* = 2
*M**_r_* = 1094.14	*F*(000) = 1112
Triclinic, *P*1	*D*_x_ = 1.586 Mg m^−^^3^
Hall symbol: -P 1	Melting point: 463 K
*a* = 11.0258 (3) Å	Mo *K*α radiation, λ = 0.71073 Å
*b* = 13.8500 (4) Å	Cell parameters from 31068 reflections
*c* = 16.0177 (4) Å	θ = 2.9–27.5°
α = 74.074 (1)°	µ = 1.29 mm^−^^1^
β = 89.742 (1)°	*T* = 150 K
γ = 77.291 (1)°	Plate, colourless
*V* = 2290.44 (11) Å^3^	0.30 × 0.25 × 0.10 mm

### Data collection

**Table:**

Nonius Kappa CCD diffractometer	10394 independent reflections
Radiation source: fine-focus sealed tube	8145 reflections with *I* > 2σ(*I*)
Graphite monochromator	*R*_int_ = 0.060
525 1.1 degree images with φ and ω scans	θ_max_ = 27.5°, θ_min_ = 3.1°
Absorption correction: multi-scan (*SORTAV*; Blessing, 1995)	*h* = −14→14
*T*_min_ = 0.699, *T*_max_ = 0.882	*k* = −17→17
35391 measured reflections	*l* = −20→20

### Refinement

**Table:**

Refinement on *F*^2^	Primary atom site location: structure-invariant direct methods
Least-squares matrix: full	Secondary atom site location: difference Fourier map
*R*[*F*^2^ > 2σ(*F*^2^)] = 0.053	Hydrogen site location: inferred from neighbouring sites
*wR*(*F*^2^) = 0.142	H atoms treated by a mixture of independent and constrained refinement
*S* = 1.05	*w* = 1/[σ^2^(*F*_o_^2^) + (0.0765*P*)^2^ + 5.2512*P*] where *P* = (*F*_o_^2^ + 2*F*_c_^2^)/3
10394 reflections	(Δ/σ)_max_ = 0.001
570 parameters	Δρ_max_ = 2.73 e Å^−^^3^
4 restraints	Δρ_min_ = −1.93 e Å^−^^3^
0 constraints	

### Fractional atomic coordinates and isotropic or equivalent isotropic displacement parameters (Å^2^)

**Table:**

	*x*	*y*	*z*	*U*_iso_*/*U*_eq_	Occ. (<1)
Sn1	0.60466 (3)	−0.00706 (2)	0.145389 (18)	0.01988 (10)	
Sn2	1.09960 (3)	0.00860 (2)	0.353582 (18)	0.02054 (10)	
P1	0.66865 (10)	0.05723 (9)	−0.07463 (7)	0.0202 (2)	
P2	0.89510 (10)	0.05951 (9)	0.16939 (7)	0.0211 (2)	
P3	0.80107 (10)	0.05415 (10)	0.42447 (7)	0.0216 (2)	
P4	1.42610 (11)	−0.06359 (10)	0.32974 (7)	0.0225 (2)	
O1	0.7119 (3)	0.0116 (3)	0.0204 (2)	0.0240 (7)	
O2	0.5310 (3)	0.0664 (3)	−0.0920 (2)	0.0252 (7)	
O3	0.7537 (3)	−0.0124 (2)	−0.1238 (2)	0.0257 (7)	
H3	0.732 (6)	0.007 (5)	−0.1791 (16)	0.06 (2)*	
O4	0.7698 (3)	0.0443 (3)	0.2040 (2)	0.0251 (7)	
O5	1.0042 (3)	0.0149 (3)	0.2355 (2)	0.0274 (7)	
O6	0.9268 (3)	0.0072 (3)	0.0935 (2)	0.0298 (8)	
H6	0.856 (3)	0.003 (4)	0.074 (4)	0.036*	
O7	0.9299 (3)	0.0723 (3)	0.4037 (2)	0.0276 (7)	
O8	0.7875 (3)	0.0056 (3)	0.5202 (2)	0.0261 (7)	
O9	0.7634 (3)	−0.0200 (3)	0.3762 (2)	0.0288 (8)	
H9	0.774 (5)	−0.014 (4)	0.3214 (15)	0.035*	
O10	1.2932 (3)	−0.0490 (3)	0.2950 (2)	0.0256 (7)	
O11	1.5111 (3)	−0.0171 (3)	0.2644 (2)	0.0265 (7)	
O12	1.4275 (3)	−0.0133 (3)	0.4068 (2)	0.0306 (8)	
H12	1.358 (3)	−0.004 (5)	0.435 (4)	0.047 (18)*	
C1	0.5157 (4)	0.1500 (4)	0.0917 (3)	0.0272 (10)	
H1A	0.5813	0.1896	0.0779	0.033*	
H1B	0.4719	0.1565	0.0360	0.033*	
C2	0.4232 (5)	0.2014 (4)	0.1454 (4)	0.0394 (13)	
H2A	0.4679	0.2043	0.1977	0.047*	
H2B	0.3617	0.1588	0.1650	0.047*	
C3	0.3536 (6)	0.3111 (5)	0.0954 (4)	0.0504 (16)	
H3A	0.3141	0.3091	0.0407	0.060*	
H3B	0.2866	0.3363	0.1308	0.060*	
C4	0.4383 (7)	0.3856 (5)	0.0741 (5)	0.063 (2)	
H4A	0.4819	0.3845	0.1276	0.094*	
H4B	0.3887	0.4553	0.0469	0.094*	
H4C	0.4994	0.3652	0.0338	0.094*	
C5	0.7103 (5)	−0.1601 (4)	0.1952 (3)	0.0286 (10)	
H5A	0.7751	−0.1597	0.2376	0.034*	
H5B	0.6544	−0.2022	0.2277	0.034*	
C6	0.7747 (5)	−0.2147 (4)	0.1303 (4)	0.0369 (12)	
H6A	0.7135	−0.2072	0.0825	0.044*	
H6B	0.8418	−0.1809	0.1046	0.044*	
C7	0.8300 (5)	−0.3290 (4)	0.1724 (4)	0.0430 (14)	
H7A	0.8868	−0.3559	0.1318	0.052*	
H7B	0.8805	−0.3361	0.2256	0.052*	
C8	0.7361 (6)	−0.3947 (5)	0.1965 (5)	0.0573 (18)	
H8A	0.6862	−0.3749	0.2424	0.086*	
H8B	0.7797	−0.4674	0.2174	0.086*	
H8C	0.6815	−0.3847	0.1453	0.086*	
C9	1.0962 (5)	−0.1487 (4)	0.4105 (3)	0.0289 (10)	
H9A	1.1828	−0.1878	0.4289	0.035*	
H9B	1.0491	−0.1531	0.4636	0.035*	
C10	1.0403 (6)	−0.2029 (4)	0.3545 (4)	0.0444 (14)	
H10A	1.0965	−0.2124	0.3076	0.053*	
H10B	0.9595	−0.1585	0.3269	0.053*	
C11	1.0199 (8)	−0.3085 (6)	0.4069 (5)	0.066 (2)	
H11A	0.9689	−0.2995	0.4564	0.080*	
H11B	0.9724	−0.3351	0.3693	0.080*	
C12	1.1337 (10)	−0.3830 (7)	0.4396 (8)	0.102 (4)	
H12A	1.1834	−0.3942	0.3908	0.153*	
H12B	1.1147	−0.4482	0.4728	0.153*	
H12C	1.1807	−0.3577	0.4775	0.153*	
C13	1.1316 (5)	0.1580 (4)	0.3025 (3)	0.0323 (11)	
H13A	1.2230	0.1497	0.3013	0.039*	0.49 (2)
H13B	1.0985	0.1822	0.2412	0.039*	0.49 (2)
H13C	1.2090	0.1515	0.2709	0.039*	0.51 (2)
H13D	1.0626	0.1984	0.2591	0.039*	0.51 (2)
C14	1.087 (2)	0.2390 (14)	0.3390 (18)	0.061 (7)	0.49 (2)
H14A	1.1296	0.2207	0.3974	0.073*	0.49 (2)
H14B	0.9974	0.2431	0.3474	0.073*	0.49 (2)
C15	1.1017 (17)	0.3441 (13)	0.2907 (13)	0.040 (4)	0.49 (2)
H15A	1.0524	0.3655	0.2346	0.048*	0.49 (2)
H15B	1.1903	0.3390	0.2773	0.048*	0.49 (2)
C16	1.064 (3)	0.4296 (12)	0.3353 (15)	0.083 (8)	0.49 (2)
H16A	0.9727	0.4530	0.3307	0.125*	0.49 (2)
H16B	1.1009	0.4875	0.3070	0.125*	0.49 (2)
H16C	1.0929	0.4031	0.3967	0.125*	0.49 (2)
C14A	1.1434 (16)	0.2205 (13)	0.3656 (10)	0.037 (4)	0.51 (2)
H14C	1.0743	0.2165	0.4051	0.044*	0.51 (2)
H14D	1.2223	0.1882	0.4015	0.044*	0.51 (2)
C15A	1.1416 (17)	0.3335 (14)	0.3234 (14)	0.043 (4)	0.51 (2)
H15C	1.1809	0.3599	0.3653	0.051*	0.51 (2)
H15D	1.1932	0.3384	0.2723	0.051*	0.51 (2)
C16A	1.0160 (13)	0.4011 (14)	0.2946 (13)	0.065 (6)	0.51 (2)
H16D	0.9770	0.3774	0.2515	0.097*	0.51 (2)
H16E	1.0237	0.4722	0.2686	0.097*	0.51 (2)
H16F	0.9644	0.3986	0.3448	0.097*	0.51 (2)
C18	0.8176 (5)	0.1993 (4)	−0.1217 (4)	0.0385 (13)	
H18	0.8864	0.1415	−0.1097	0.046*	
C19	0.8366 (6)	0.2982 (5)	−0.1480 (5)	0.0550 (18)	
H19	0.9190	0.3083	−0.1539	0.066*	
C20	0.7368 (6)	0.3827 (5)	−0.1659 (4)	0.0501 (16)	
H20	0.7509	0.4504	−0.1846	0.060*	
C21	0.6185 (6)	0.3688 (4)	−0.1567 (4)	0.0464 (15)	
H21	0.5503	0.4270	−0.1684	0.056*	
C22	0.5972 (5)	0.2706 (4)	−0.1305 (4)	0.0363 (12)	
H22	0.5143	0.2616	−0.1246	0.044*	
C23	0.6961 (4)	0.1852 (4)	−0.1128 (3)	0.0230 (9)	
C24	0.8867 (5)	0.1947 (4)	0.1239 (3)	0.0286 (10)	
C25	0.7865 (5)	0.2678 (4)	0.1385 (4)	0.0388 (13)	
H25	0.7199	0.2465	0.1709	0.047*	
C26	0.7844 (6)	0.3719 (5)	0.1056 (5)	0.0556 (18)	
H26	0.7146	0.4220	0.1136	0.067*	
C27	0.8827 (7)	0.4030 (5)	0.0614 (5)	0.0571 (18)	
H27	0.8820	0.4743	0.0405	0.068*	
C28	0.9822 (6)	0.3304 (5)	0.0477 (4)	0.0528 (17)	
H28	1.0495	0.3522	0.0165	0.063*	
C29	0.9856 (5)	0.2271 (5)	0.0784 (4)	0.0402 (13)	
H29	1.0550	0.1778	0.0687	0.048*	
C30	0.6932 (4)	0.1762 (4)	0.3882 (3)	0.0264 (10)	
C31	0.7309 (6)	0.2674 (4)	0.3687 (4)	0.0451 (14)	
H31	0.8171	0.2667	0.3726	0.054*	
C32	0.6441 (8)	0.3607 (5)	0.3434 (5)	0.066 (2)	
H32	0.6707	0.4237	0.3295	0.079*	
C33	0.5190 (7)	0.3615 (5)	0.3384 (5)	0.060 (2)	
H33	0.4595	0.4254	0.3207	0.072*	
C34	0.4804 (6)	0.2714 (5)	0.3586 (5)	0.0534 (17)	
H34	0.3939	0.2729	0.3553	0.064*	
C35	0.5654 (5)	0.1783 (5)	0.3838 (4)	0.0386 (13)	
H35	0.5378	0.1157	0.3981	0.046*	
C36	1.4922 (4)	−0.1989 (4)	0.3735 (3)	0.0288 (10)	
C37	1.4313 (5)	−0.2721 (4)	0.3611 (4)	0.0389 (13)	
H37	1.3520	−0.2509	0.3304	0.047*	
C38	1.4865 (6)	−0.3759 (5)	0.3935 (4)	0.0491 (15)	
H38	1.4442	−0.4261	0.3860	0.059*	
C39	1.6026 (6)	−0.4071 (5)	0.4366 (4)	0.0530 (17)	
H39	1.6402	−0.4784	0.4583	0.064*	
C40	1.6642 (6)	−0.3346 (5)	0.4481 (4)	0.0511 (16)	
H40	1.7441	−0.3563	0.4779	0.061*	
C41	1.6103 (5)	−0.2309 (5)	0.4165 (4)	0.0400 (13)	
H41	1.6534	−0.1813	0.4239	0.048*	

### Atomic displacement parameters (Å^2^)

**Table:**

	*U*^11^	*U*^22^	*U*^33^	*U*^12^	*U*^13^	*U*^23^
Sn1	0.01548 (16)	0.02907 (18)	0.01582 (16)	−0.00556 (12)	0.00436 (11)	−0.00712 (12)
Sn2	0.01641 (16)	0.03061 (18)	0.01525 (16)	−0.00626 (12)	0.00486 (11)	−0.00684 (12)
P1	0.0162 (5)	0.0304 (6)	0.0150 (5)	−0.0061 (4)	0.0047 (4)	−0.0075 (5)
P2	0.0178 (5)	0.0315 (6)	0.0146 (5)	−0.0058 (5)	0.0038 (4)	−0.0071 (5)
P3	0.0176 (5)	0.0327 (6)	0.0160 (5)	−0.0066 (5)	0.0052 (4)	−0.0086 (5)
P4	0.0197 (5)	0.0338 (6)	0.0146 (5)	−0.0073 (5)	0.0058 (4)	−0.0068 (5)
O1	0.0211 (16)	0.0335 (17)	0.0171 (15)	−0.0070 (13)	0.0038 (12)	−0.0060 (13)
O2	0.0196 (15)	0.0360 (18)	0.0211 (16)	−0.0056 (13)	0.0002 (13)	−0.0104 (14)
O3	0.0261 (17)	0.0300 (18)	0.0194 (16)	−0.0001 (14)	0.0019 (13)	−0.0091 (14)
O4	0.0200 (16)	0.0384 (19)	0.0189 (16)	−0.0096 (14)	0.0054 (12)	−0.0093 (14)
O5	0.0247 (17)	0.0393 (19)	0.0187 (16)	−0.0078 (14)	0.0039 (13)	−0.0086 (14)
O6	0.0268 (18)	0.043 (2)	0.0231 (17)	−0.0060 (16)	0.0053 (14)	−0.0168 (15)
O7	0.0203 (16)	0.0413 (19)	0.0243 (17)	−0.0092 (14)	0.0130 (13)	−0.0127 (15)
O8	0.0205 (16)	0.0383 (19)	0.0174 (15)	−0.0051 (14)	0.0072 (12)	−0.0058 (13)
O9	0.0354 (19)	0.0348 (19)	0.0202 (16)	−0.0114 (15)	0.0101 (14)	−0.0116 (15)
O10	0.0209 (16)	0.0365 (19)	0.0205 (16)	−0.0073 (14)	0.0056 (13)	−0.0092 (14)
O11	0.0243 (16)	0.0390 (19)	0.0168 (16)	−0.0081 (14)	0.0115 (13)	−0.0083 (14)
O12	0.0263 (18)	0.048 (2)	0.0225 (17)	−0.0125 (16)	0.0069 (14)	−0.0147 (15)
C1	0.026 (2)	0.031 (3)	0.022 (2)	−0.0052 (19)	0.0074 (19)	−0.0047 (19)
C2	0.045 (3)	0.032 (3)	0.036 (3)	−0.002 (2)	0.019 (2)	−0.008 (2)
C3	0.048 (4)	0.045 (3)	0.052 (4)	0.002 (3)	0.019 (3)	−0.014 (3)
C4	0.071 (5)	0.038 (4)	0.076 (5)	−0.006 (3)	0.013 (4)	−0.013 (3)
C5	0.029 (2)	0.035 (3)	0.021 (2)	−0.006 (2)	0.0024 (19)	−0.007 (2)
C6	0.031 (3)	0.040 (3)	0.040 (3)	−0.007 (2)	0.013 (2)	−0.013 (2)
C7	0.036 (3)	0.038 (3)	0.054 (4)	−0.003 (2)	0.011 (3)	−0.016 (3)
C8	0.050 (4)	0.043 (4)	0.080 (5)	−0.011 (3)	0.018 (4)	−0.018 (3)
C9	0.030 (3)	0.033 (3)	0.023 (2)	−0.009 (2)	0.0009 (19)	−0.006 (2)
C10	0.060 (4)	0.038 (3)	0.040 (3)	−0.018 (3)	−0.002 (3)	−0.013 (3)
C11	0.072 (5)	0.062 (5)	0.070 (5)	−0.027 (4)	0.001 (4)	−0.018 (4)
C12	0.104 (8)	0.062 (5)	0.131 (9)	−0.022 (5)	−0.028 (7)	−0.009 (6)
C13	0.038 (3)	0.037 (3)	0.022 (2)	−0.013 (2)	0.004 (2)	−0.004 (2)
C14	0.053 (12)	0.043 (9)	0.095 (18)	−0.022 (9)	0.049 (12)	−0.024 (10)
C15	0.031 (9)	0.031 (7)	0.057 (11)	−0.002 (7)	−0.008 (6)	−0.012 (7)
C16	0.14 (2)	0.044 (9)	0.082 (14)	−0.030 (10)	0.035 (13)	−0.040 (9)
C14A	0.032 (8)	0.049 (8)	0.033 (7)	−0.014 (7)	−0.001 (6)	−0.012 (6)
C15A	0.033 (9)	0.044 (8)	0.059 (12)	−0.018 (8)	0.016 (7)	−0.020 (9)
C16A	0.039 (8)	0.061 (11)	0.081 (13)	−0.005 (7)	0.010 (7)	−0.004 (9)
C18	0.026 (3)	0.044 (3)	0.046 (3)	−0.013 (2)	0.006 (2)	−0.010 (3)
C19	0.040 (3)	0.052 (4)	0.075 (5)	−0.027 (3)	0.013 (3)	−0.009 (3)
C20	0.063 (4)	0.037 (3)	0.055 (4)	−0.024 (3)	0.014 (3)	−0.009 (3)
C21	0.049 (4)	0.033 (3)	0.053 (4)	−0.008 (3)	0.008 (3)	−0.006 (3)
C22	0.028 (3)	0.037 (3)	0.041 (3)	−0.006 (2)	0.007 (2)	−0.007 (2)
C23	0.022 (2)	0.030 (2)	0.019 (2)	−0.0102 (18)	0.0042 (17)	−0.0079 (18)
C24	0.028 (2)	0.032 (3)	0.026 (2)	−0.010 (2)	−0.0024 (19)	−0.005 (2)
C25	0.031 (3)	0.038 (3)	0.050 (3)	−0.010 (2)	0.005 (2)	−0.014 (3)
C26	0.050 (4)	0.034 (3)	0.080 (5)	−0.003 (3)	−0.004 (3)	−0.017 (3)
C27	0.064 (4)	0.036 (3)	0.070 (5)	−0.022 (3)	0.002 (4)	−0.005 (3)
C28	0.056 (4)	0.052 (4)	0.050 (4)	−0.026 (3)	0.010 (3)	−0.002 (3)
C29	0.038 (3)	0.043 (3)	0.037 (3)	−0.012 (2)	0.009 (2)	−0.004 (2)
C30	0.026 (2)	0.033 (3)	0.019 (2)	−0.0016 (19)	0.0037 (18)	−0.0097 (19)
C31	0.041 (3)	0.036 (3)	0.057 (4)	−0.013 (3)	0.007 (3)	−0.008 (3)
C32	0.086 (6)	0.033 (3)	0.073 (5)	−0.011 (3)	0.013 (4)	−0.009 (3)
C33	0.066 (5)	0.044 (4)	0.053 (4)	0.024 (3)	−0.010 (3)	−0.014 (3)
C34	0.039 (3)	0.050 (4)	0.063 (4)	0.009 (3)	−0.009 (3)	−0.017 (3)
C35	0.028 (3)	0.044 (3)	0.045 (3)	−0.006 (2)	0.000 (2)	−0.015 (3)
C36	0.027 (2)	0.037 (3)	0.020 (2)	−0.007 (2)	0.0071 (19)	−0.006 (2)
C37	0.035 (3)	0.041 (3)	0.040 (3)	−0.007 (2)	0.006 (2)	−0.012 (2)
C38	0.054 (4)	0.032 (3)	0.059 (4)	−0.013 (3)	0.011 (3)	−0.007 (3)
C39	0.057 (4)	0.037 (3)	0.050 (4)	0.005 (3)	0.012 (3)	−0.001 (3)
C40	0.044 (3)	0.051 (4)	0.042 (3)	0.002 (3)	0.000 (3)	0.004 (3)
C41	0.040 (3)	0.043 (3)	0.031 (3)	−0.006 (2)	0.002 (2)	−0.002 (2)

### Geometric parameters (Å, º)

**Table:**

Sn1—C1	2.116 (5)	C12—H12C	0.9800
Sn1—C5	2.117 (5)	C13—C14	1.398 (19)
Sn1—O2^i^	2.140 (3)	C13—C14A	1.522 (16)
Sn1—O11^ii^	2.149 (3)	C13—H13A	0.9900
Sn1—O1	2.303 (3)	C13—H13B	0.9900
Sn1—O4	2.378 (3)	C13—H13C	0.9900
Sn2—C13	2.113 (5)	C13—H13D	0.9900
Sn2—C9	2.127 (5)	C14—C15	1.49 (3)
Sn2—O7	2.138 (3)	C14—H14A	0.9900
Sn2—O5	2.139 (3)	C14—H14B	0.9900
Sn2—O8^iii^	2.317 (3)	C15—C16	1.53 (3)
Sn2—O10	2.391 (3)	C15—H15A	0.9900
P1—O1	1.513 (3)	C15—H15B	0.9900
P1—O2	1.516 (3)	C16—H16A	0.9800
P1—O3	1.563 (3)	C16—H16B	0.9800
P1—C23	1.802 (5)	C16—H16C	0.9800
P2—O5	1.509 (3)	C14A—C15A	1.52 (2)
P2—O4	1.522 (3)	C14A—H14C	0.9900
P2—O6	1.580 (4)	C14A—H14D	0.9900
P2—C24	1.795 (5)	C15A—C16A	1.49 (2)
P3—O7	1.517 (3)	C15A—H15C	0.9900
P3—O8	1.518 (3)	C15A—H15D	0.9900
P3—O9	1.566 (4)	C16A—H16D	0.9800
P3—C30	1.786 (5)	C16A—H16E	0.9800
P4—O11	1.508 (3)	C16A—H16F	0.9800
P4—O10	1.521 (3)	C18—C19	1.380 (8)
P4—O12	1.577 (4)	C18—C23	1.398 (7)
P4—C36	1.794 (5)	C18—H18	0.9500
O2—Sn1^i^	2.140 (3)	C19—C20	1.383 (9)
O3—H3	0.87 (2)	C19—H19	0.9500
O6—H6	0.861 (19)	C20—C21	1.361 (9)
O8—Sn2^iii^	2.317 (3)	C20—H20	0.9500
O9—H9	0.867 (19)	C21—C22	1.382 (8)
O11—Sn1^iv^	2.149 (3)	C21—H21	0.9500
O12—H12	0.88 (2)	C22—C23	1.385 (7)
C1—C2	1.514 (7)	C22—H22	0.9500
C1—H1A	0.9900	C24—C25	1.391 (7)
C1—H1B	0.9900	C24—C29	1.397 (7)
C2—C3	1.537 (8)	C25—C26	1.388 (8)
C2—H2A	0.9900	C25—H25	0.9500
C2—H2B	0.9900	C26—C27	1.376 (10)
C3—C4	1.511 (9)	C26—H26	0.9500
C3—H3A	0.9900	C27—C28	1.377 (10)
C3—H3B	0.9900	C27—H27	0.9500
C4—H4A	0.9800	C28—C29	1.371 (8)
C4—H4B	0.9800	C28—H28	0.9500
C4—H4C	0.9800	C29—H29	0.9500
C5—C6	1.531 (7)	C30—C31	1.371 (7)
C5—H5A	0.9900	C30—C35	1.405 (7)
C5—H5B	0.9900	C31—C32	1.386 (9)
C6—C7	1.525 (8)	C31—H31	0.9500
C6—H6A	0.9900	C32—C33	1.379 (11)
C6—H6B	0.9900	C32—H32	0.9500
C7—C8	1.506 (9)	C33—C34	1.362 (10)
C7—H7A	0.9900	C33—H33	0.9500
C7—H7B	0.9900	C34—C35	1.375 (8)
C8—H8A	0.9800	C34—H34	0.9500
C8—H8B	0.9800	C35—H35	0.9500
C8—H8C	0.9800	C36—C37	1.389 (8)
C9—C10	1.524 (7)	C36—C41	1.398 (7)
C9—H9A	0.9900	C37—C38	1.385 (8)
C9—H9B	0.9900	C37—H37	0.9500
C10—C11	1.537 (9)	C38—C39	1.379 (9)
C10—H10A	0.9900	C38—H38	0.9500
C10—H10B	0.9900	C39—C40	1.381 (10)
C11—C12	1.433 (11)	C39—H39	0.9500
C11—H11A	0.9900	C40—C41	1.378 (8)
C11—H11B	0.9900	C40—H40	0.9500
C12—H12A	0.9800	C41—H41	0.9500
C12—H12B	0.9800		
			
C1—Sn1—C5	174.37 (18)	C12—C11—H11B	108.9
C1—Sn1—O2^i^	95.48 (16)	C10—C11—H11B	108.9
C5—Sn1—O2^i^	88.55 (16)	H11A—C11—H11B	107.7
C1—Sn1—O11^ii^	92.86 (15)	C11—C12—H12A	109.5
C5—Sn1—O11^ii^	90.93 (16)	C11—C12—H12B	109.5
O2^i^—Sn1—O11^ii^	91.82 (12)	H12A—C12—H12B	109.5
C1—Sn1—O1	85.51 (15)	C11—C12—H12C	109.5
C5—Sn1—O1	90.44 (15)	H12A—C12—H12C	109.5
O2^i^—Sn1—O1	91.96 (12)	H12B—C12—H12C	109.5
O11^ii^—Sn1—O1	176.01 (12)	C14—C13—Sn2	123.2 (8)
C1—Sn1—O4	89.40 (16)	C14A—C13—Sn2	118.2 (7)
C5—Sn1—O4	86.40 (16)	C14—C13—H13A	106.5
O2^i^—Sn1—O4	174.42 (12)	C14A—C13—H13A	83.6
O11^ii^—Sn1—O4	90.65 (12)	Sn2—C13—H13A	106.5
O1—Sn1—O4	85.70 (11)	C14—C13—H13B	106.5
C13—Sn2—C9	171.5 (2)	C14A—C13—H13B	129.2
C13—Sn2—O7	90.09 (17)	Sn2—C13—H13B	106.5
C9—Sn2—O7	96.29 (16)	H13A—C13—H13B	106.5
C13—Sn2—O5	92.65 (17)	C14—C13—H13C	123.3
C9—Sn2—O5	92.77 (16)	C14A—C13—H13C	107.8
O7—Sn2—O5	91.74 (12)	Sn2—C13—H13C	107.8
C13—Sn2—O8^iii^	88.59 (16)	H13B—C13—H13C	78.2
C9—Sn2—O8^iii^	85.65 (15)	C14—C13—H13D	80.8
O7—Sn2—O8^iii^	91.44 (12)	C14A—C13—H13D	107.8
O5—Sn2—O8^iii^	176.59 (12)	Sn2—C13—H13D	107.8
C13—Sn2—O10	85.28 (17)	H13A—C13—H13D	132.2
C9—Sn2—O10	88.24 (16)	H13C—C13—H13D	107.1
O7—Sn2—O10	175.30 (12)	C13—C14—C15	118.2 (15)
O5—Sn2—O10	89.30 (12)	C13—C14—H14A	107.8
O8^iii^—Sn2—O10	87.63 (11)	C15—C14—H14A	107.8
O1—P1—O2	113.49 (18)	C13—C14—H14B	107.8
O1—P1—O3	105.59 (18)	C15—C14—H14B	107.8
O2—P1—O3	113.04 (19)	H14A—C14—H14B	107.1
O1—P1—C23	109.6 (2)	C14—C15—C16	117.4 (16)
O2—P1—C23	107.0 (2)	C14—C15—H15A	108.0
O3—P1—C23	107.9 (2)	C16—C15—H15A	108.0
O5—P2—O4	115.01 (18)	C14—C15—H15B	108.0
O5—P2—O6	106.31 (19)	C16—C15—H15B	108.0
O4—P2—O6	110.19 (19)	H15A—C15—H15B	107.2
O5—P2—C24	108.7 (2)	C15A—C14A—C13	115.1 (12)
O4—P2—C24	109.3 (2)	C15A—C14A—H14C	108.5
O6—P2—C24	106.9 (2)	C13—C14A—H14C	108.5
O7—P3—O8	113.94 (19)	C15A—C14A—H14D	108.5
O7—P3—O9	112.23 (19)	C13—C14A—H14D	108.5
O8—P3—O9	105.37 (19)	H14C—C14A—H14D	107.5
O7—P3—C30	107.3 (2)	C16A—C15A—C14A	115.1 (13)
O8—P3—C30	109.8 (2)	C16A—C15A—H15C	108.5
O9—P3—C30	108.1 (2)	C14A—C15A—H15C	108.5
O11—P4—O10	115.26 (18)	C16A—C15A—H15D	108.5
O11—P4—O12	106.10 (19)	C14A—C15A—H15D	108.5
O10—P4—O12	110.47 (19)	H15C—C15A—H15D	107.5
O11—P4—C36	108.9 (2)	C15A—C16A—H16D	109.5
O10—P4—C36	108.9 (2)	C15A—C16A—H16E	109.5
O12—P4—C36	106.9 (2)	H16D—C16A—H16E	109.5
P1—O1—Sn1	131.87 (18)	C15A—C16A—H16F	109.5
P1—O2—Sn1^i^	145.6 (2)	H16D—C16A—H16F	109.5
P1—O3—H3	111 (5)	H16E—C16A—H16F	109.5
P2—O4—Sn1	131.42 (18)	C19—C18—C23	119.2 (5)
P2—O5—Sn2	151.1 (2)	C19—C18—H18	120.4
P2—O6—H6	105 (4)	C23—C18—H18	120.4
P3—O7—Sn2	141.8 (2)	C18—C19—C20	120.7 (6)
P3—O8—Sn2^iii^	133.47 (19)	C18—C19—H19	119.6
P3—O9—H9	123 (4)	C20—C19—H19	119.6
P4—O10—Sn2	130.14 (19)	C21—C20—C19	120.0 (6)
P4—O11—Sn1^iv^	151.1 (2)	C21—C20—H20	120.0
P4—O12—H12	117 (4)	C19—C20—H20	120.0
C2—C1—Sn1	118.2 (3)	C20—C21—C22	120.3 (6)
C2—C1—H1A	107.8	C20—C21—H21	119.8
Sn1—C1—H1A	107.8	C22—C21—H21	119.8
C2—C1—H1B	107.8	C21—C22—C23	120.3 (5)
Sn1—C1—H1B	107.8	C21—C22—H22	119.8
H1A—C1—H1B	107.1	C23—C22—H22	119.8
C1—C2—C3	113.3 (4)	C22—C23—C18	119.4 (5)
C1—C2—H2A	108.9	C22—C23—P1	120.3 (4)
C3—C2—H2A	108.9	C18—C23—P1	120.2 (4)
C1—C2—H2B	108.9	C25—C24—C29	119.5 (5)
C3—C2—H2B	108.9	C25—C24—P2	120.6 (4)
H2A—C2—H2B	107.7	C29—C24—P2	119.7 (4)
C4—C3—C2	112.6 (5)	C26—C25—C24	119.6 (5)
C4—C3—H3A	109.1	C26—C25—H25	120.2
C2—C3—H3A	109.1	C24—C25—H25	120.2
C4—C3—H3B	109.1	C27—C26—C25	120.4 (6)
C2—C3—H3B	109.1	C27—C26—H26	119.8
H3A—C3—H3B	107.8	C25—C26—H26	119.8
C3—C4—H4A	109.5	C26—C27—C28	119.8 (6)
C3—C4—H4B	109.5	C26—C27—H27	120.1
H4A—C4—H4B	109.5	C28—C27—H27	120.1
C3—C4—H4C	109.5	C29—C28—C27	121.0 (6)
H4A—C4—H4C	109.5	C29—C28—H28	119.5
H4B—C4—H4C	109.5	C27—C28—H28	119.5
C6—C5—Sn1	117.8 (3)	C28—C29—C24	119.7 (6)
C6—C5—H5A	107.9	C28—C29—H29	120.1
Sn1—C5—H5A	107.9	C24—C29—H29	120.2
C6—C5—H5B	107.9	C31—C30—C35	119.2 (5)
Sn1—C5—H5B	107.9	C31—C30—P3	122.1 (4)
H5A—C5—H5B	107.2	C35—C30—P3	118.6 (4)
C7—C6—C5	112.4 (5)	C30—C31—C32	120.4 (6)
C7—C6—H6A	109.1	C30—C31—H31	119.8
C5—C6—H6A	109.1	C32—C31—H31	119.8
C7—C6—H6B	109.1	C33—C32—C31	119.7 (6)
C5—C6—H6B	109.1	C33—C32—H32	120.2
H6A—C6—H6B	107.9	C31—C32—H32	120.2
C8—C7—C6	115.0 (5)	C34—C33—C32	120.4 (6)
C8—C7—H7A	108.5	C34—C33—H33	119.8
C6—C7—H7A	108.5	C32—C33—H33	119.8
C8—C7—H7B	108.5	C33—C34—C35	120.6 (6)
C6—C7—H7B	108.5	C33—C34—H34	119.7
H7A—C7—H7B	107.5	C35—C34—H34	119.7
C7—C8—H8A	109.5	C34—C35—C30	119.7 (6)
C7—C8—H8B	109.5	C34—C35—H35	120.2
H8A—C8—H8B	109.5	C30—C35—H35	120.2
C7—C8—H8C	109.5	C37—C36—C41	119.5 (5)
H8A—C8—H8C	109.5	C37—C36—P4	121.2 (4)
H8B—C8—H8C	109.5	C41—C36—P4	119.2 (4)
C10—C9—Sn2	117.3 (3)	C38—C37—C36	119.7 (5)
C10—C9—H9A	108.0	C38—C37—H37	120.1
Sn2—C9—H9A	108.0	C36—C37—H37	120.1
C10—C9—H9B	108.0	C39—C38—C37	120.4 (6)
Sn2—C9—H9B	108.0	C39—C38—H38	119.8
H9A—C9—H9B	107.2	C37—C38—H38	119.8
C9—C10—C11	112.7 (5)	C38—C39—C40	120.0 (6)
C9—C10—H10A	109.0	C38—C39—H39	120.0
C11—C10—H10A	109.0	C40—C39—H39	120.0
C9—C10—H10B	109.0	C41—C40—C39	120.3 (6)
C11—C10—H10B	109.0	C41—C40—H40	119.9
H10A—C10—H10B	107.8	C39—C40—H40	119.9
C12—C11—C10	113.3 (7)	C40—C41—C36	119.9 (6)
C12—C11—H11A	108.9	C40—C41—H41	120.0
C10—C11—H11A	108.9	C36—C41—H41	120.0

Symmetry codes: (i) −*x*+1, −*y*, −*z*; (ii) *x*−1, *y*, *z*; (iii) −*x*+2, −*y*, −*z*+1; (iv) *x*+1, *y*, *z*.

### Hydrogen-bond geometry (Å, º)

**Table:**

*D*—H···*A*	*D*—H	H···*A*	*D*···*A*	*D*—H···*A*
O3—H3···O10^v^	0.87 (2)	1.80 (1)	2.656 (5)	172 (6)
O6—H6···O1	0.86 (2)	1.77 (1)	2.628 (5)	172 (6)
O9—H9···O4	0.87 (2)	1.83 (2)	2.662 (5)	159 (6)
O12—H12···O8^iii^	0.88 (2)	1.78 (2)	2.633 (5)	168 (6)

Symmetry codes: (iii) −*x*+2, −*y*, −*z*+1; (v) −*x*+2, −*y*, −*z*.
